# A New Era for Maternal and Child Nutrition Implementation Science Research and Program Evaluation

**DOI:** 10.1111/mcn.70012

**Published:** 2025-03-06

**Authors:** Rafael Pérez‐Escamilla, Victoria H. Moran

**Affiliations:** ^1^ Department of Social and Behavioral Sciences Yale School of Public Health New Haven Connecticut USA; ^2^ Maternal, Paternal & Infant Nutrition & Nurture Unit (MAINN) University of Central Lancashire Preston UK

Since its inception 2005, *Maternal & Child Nutrition* has prided itself on having been at the forefront of publishing high‐quality studies based on innovative mixed implementation science methods. This issue includes a special section on lessons learned from the Alive & Thrive (A&T) initiative implemented in low‐ and middle‐income countries located in south and southeast Asia and Sub‐Saharan Africa to improve infant, young child, and adolescent nutrition (MIYCAN) outcomes. During 2009 and 2014, A&T developed and implemented MIYCAN interventions at scale in three countries and subsequently expanded its work to six country‐specific and two regional programs, to address maternal and adolescent nutrition in the context of agriculture and social protection programs (Frongillo et al. 2025).

The A&T evidence presented in the special section of this issue and elsewhere (e.g., Menon et al. 2016; Kim et al. 2019, Sanghvi et al. 2022, 2025; Siekmans et al. 2024) highlights the great contributions that the field of implementation science in nutrition (Tumilowicz et al. 2018) has made in improving our understanding of best practices to scale up MIYCAN programs so that they are cost‐effective and sustainable in the context of complex adaptive systems (Paina and Peters 2012). This body of work leaves little doubt that effective and sustainable programs with potential for dissemination require transparent and inclusive stakeholder engagement and advocacy, and clear goals from the start to guide policy, program codesign, impact pathways, costing and financing, and quality assurance based on mixed methods process and impact evaluations, and sound technical assistance. Furthermore, this work collectively illustrates the need to guide the program codesign with sound person and family centered social and behavioral change theories and systems frameworks rooted in equity principles. These conclusions align with other MIYCAN programs such as the *Suaahara* project in Nepal (Frongillo et al. 2024), the *Becoming Breastfeeding Initiative* that has supported the development and implementation of national breastfeeding programs in 10 countries across five world regions (Pérez‐Escamilla et al. 2023), and large‐scale micronutrient initiatives (Reerink et al. 2017).

The publication of this special section signals the end of one of the richest implementation science experiences in MIYCAF. The A&T initiative has indeed generated a wealth of knowledge of scale up within countries and program dissemination across countries (i.e., scale out). Furthermore, it provides key case studies for understanding how program adaptations need to be implemented and monitored.

Moving forward it is key for researchers and program evaluators to continue expanding this work, adding more depth to our understanding of why and how program adaptations need to be made to meet the requirements of the local context(s) where they operate (Martinez‐Brockman et al. 2025). Importantly such studies need to assess if the adaptations made resulted in improvements, or not, to program operations. This knowledge is crucial for enhancing the success of program dissemination across contexts (Bradley et al. 2012). Innovative research is needed to identify community‐engaged codesign best practices (Segura‐Pérez et al. 2025) to ensure that new MIYCAN programs, or those that are in place but need to be re‐designed, have a much better chance for scale‐up and sustainability.

We would like to end by encouraging funders to prioritize and expand resources allocation to scale up research and program evaluation across settings. This will be essential for shortening the amount of time that it takes to translate lifesaving MIYCF evidence‐based knowledge into practice.

## References

[mcn70012-bib-0001] Bradley, E. H. , L. A. Curry , L. A. Taylor , et al. 2012. “A Model for Scale Up of Family Health Innovations in Low‐Income and Middle‐Income Settings: A Mixed Methods Study.” BMJ Open 2, no. 4: e000987. 10.1136/bmjopen-2012-000987. PMC3432850 22923624

[mcn70012-bib-0002] Frongillo, E. A. , J. Escobar‐DeMarco , and S. Bose . 2025. “Assessment of Four Essential Features of the Alive & Thrive Initiative to Improve Maternal, Infant, Young Child, and Adolescent Nutrition.” Maternal & Child Nutrition 21: e13800. 10.1111/mcn.13800. PMC11956034 39868492

[mcn70012-bib-0003] Frongillo, E. A. , S. Suresh , D. K. Thapa , et al. 2024. “Impact of Suaahara, an Integrated Nutrition Programme, on Maternal and Child Nutrition at Scale in Nepal.” Maternal & Child Nutrition 21: e13630. 10.1111/mcn.13630. PMC12647975 38342986

[mcn70012-bib-0004] Kim, S. S. , P. H. Nguyen , Y. Yohannes , et al. 2019. “Behavior Change Interventions Delivered Through Interpersonal Communication, Agricultural Activities, Community Mobilization, and Mass Media Increase Complementary Feeding Practices and Reduce Child Stunting in Ethiopia.” Journal of Nutrition 149, no. 8: 1470–1481. 10.1093/jn/nxz087. 31165869 PMC6686053

[mcn70012-bib-0005] Martinez‐Brockman, J. L. , J. R. Granner , B. Buchanan , et al. 2025. “Evaluation and Adaptation of a Two‐Way Text Messaging Intervention in the WIC Breastfeeding Peer Counseling Program: A Qualitative Analysis.” PLoS One 20, no. 1: e0313779. 10.1371/journal.pone.0313779. 39787090 PMC11717301

[mcn70012-bib-0006] Menon, P. , P. H. Nguyen , K. K. Saha , et al. 2016. “Impacts on Breastfeeding Practices of At‐Scale Strategies That Combine Intensive Interpersonal Counseling, Mass Media, and Community Mobilization: Results of Cluster‐Randomized Program Evaluations in Bangladesh and Vietnam.” PLoS Medicine 13: e1002159. 27780198 10.1371/journal.pmed.1002159 PMC5079648

[mcn70012-bib-0007] Paina, L. , and D. H. Peters . 2012. “Understanding Pathways for Scaling Up Health Services Through the Lens of Complex Adaptive Systems.” Health Policy and Planning 27, no. 5: 365–373. 10.1093/heapol/czr054. 21821667

[mcn70012-bib-0008] Pérez‐Escamilla, R. , F. C. Dykes , and S. Kendall . 2023. “Gearing to Success With National Breastfeeding Programmes: The Becoming Breastfeeding Friendly (BBF) Initiative Experience.” Supplement, Maternal & Child Nutrition 19, no. Suppl 1: e13339. 10.1111/mcn.13339. 35254735 PMC9835584

[mcn70012-bib-0009] Reerink, I. , S. M. Namaste , A. Poonawala , et al. 2017. “Experiences and Lessons Learned for Delivery of Micronutrient Powders Interventions.” Supplement, Maternal & Child Nutrition 13, no. Suppl 1: e12495. 10.1111/mcn.12495. 28960878 PMC5656897

[mcn70012-bib-0010] Sanghvi, T. , P. H. Nguyen , S. Ghosh , et al. 2022. “Process of Developing Models of Maternal Nutrition Interventions Integrated Into Antenatal Care Services in Bangladesh, Burkina Faso, Ethiopia and India.” Maternal & Child Nutrition 18: e13379. 10.1111/mcn.13379. 35698901 PMC9480954

[mcn70012-bib-0011] Sanghvi, T. G. , S. Remancus, E. A. Frongillo, et al. 2025. “Evidence‐Based Lessons From Two Decades of Implementation Research on Large Scale Complementary Feeding Programs.” Maternal & Child Nutrition: e13811. 10.1111/mcn.13811. 40018978 PMC12150138

[mcn70012-bib-0012] Segura‐Pérez, S. , A. Tristán Urrutia , A. He , et al. 2025. “Community Engaged Co‐Design and Piloting of the FOOD4MOMS Produce Prescription Program for Pregnant Latina Women.” Current Developments in Nutrition 9, no. 3: 104572. 10.1016/j.cdnut.2025.104572. 40145018 PMC11938078

[mcn70012-bib-0013] Siekmans, K. , S. Bose , J. Escobar‐DeMarco , and E. A. Frongillo . 2024. “Strengthening Nutrition Policy and Service Delivery: Lessons Learned From a Six‐Country Assessment of Alive and Thrive's Technical Assistance.” Maternal & Child Nutrition 21: e13711. 10.1111/mcn.13711. PMC11956047 39363438

[mcn70012-bib-0014] Tumilowicz, A. , M. T. Ruel , G. Pelto , et al. 2019. “Implementation Science in Nutrition: Concepts and Frameworks for an Emerging Field of Science and Practice.” Current Developments in Nutrition 3, no. 3: nzy080. 10.1093/cdn/nzy080. 30864563 PMC6400593

